# Genetic analyses place most Spanish isolates of *Beauveria bassiana *in a molecular group with word-wide distribution

**DOI:** 10.1186/1471-2180-11-84

**Published:** 2011-04-26

**Authors:** Inmaculada Garrido-Jurado, Marcela Márquez, Almudena Ortiz-Urquiza, Cándido Santiago-Álvarez, Enrique A Iturriaga, Enrique Quesada-Moraga, Enrique Monte, Rosa Hermosa

**Affiliations:** 1 Departamento de Ciencias y Recursos Agrícolas y Forestales, Universidad de Córdoba, Edificio C4 Celestino Mutis, Campus Rabanales, 14071 Córdoba, Spain; 2 Area de Genética. Departamento de Microbiología y Genética, Universidad de Salamanca, Edificio Departamental lab 324, Plaza Doctores de la Reina s/n, 37007 Salamanca, Spain; 3 Centro Hispano-Luso de Investigaciones Agrarias (CIALE), Departamento de Microbiología y Genética, Universidad de Salamanca, Río Duero 12, Campus de Villamayor, 37185 Salamanca, Spain

**Keywords:** Entomopathogenic fungi, LSU rDNA group I introns, Translation elongation factor gene, Thermal growth

## Abstract

**Background:**

The entomopathogenic anamorphic fungus *Beauveria bassiana *is currently used as a biocontrol agent (BCA) of insects. Fifty-seven *Beauveria bassiana *isolates -53 from Spain- were characterized, integrating group I intron insertion patterns at the 3'-end of the nuclear large subunit ribosomal gene (LSU rDNA) and elongation factor 1-alpha (EF1-α) phylogenetic information, in order to assess the genetic structure and diversity of this Spanish collection of *B. bassiana*.

**Results:**

Group I intron genotype analysis was based on the four highly conserved insertion sites of the LSU (Ec2653, Ec2449, Ec2066, Ec1921). Of the 16 possible combinations/genotypes, only four were detected, two of which were predominant, containing 44 and 9 members out of 57 isolates, respectively. Interestingly, the members of the latter two genotypes showed unique differences in their growth temperatures. In follow, EF1-α phylogeny served to classify most of the strains in the *B. bassiana s.s*. (*sensu stricto*) group and separate them into 5 molecular subgroups, all of which contained a group I intron belonging to the IC1 subtype at the Ec1921 position. A number of parameters such as thermal growth or origin (host, geographic location and climatic conditions) were also examined but in general no association could be found.

**Conclusion:**

Most Spanish *B. bassiana *isolates (77.2%) are grouped into a major phylogenetic subgroup with word-wide distribution. However, high phylogenetic diversity was also detected among Spanish isolates from close geographic zones with low climatic variation. In general, no correlation was observed between the molecular distribution and geographic origin or climatic characteristics where the Spanish *B. bassiana *isolates were sampled.

## Background

The anamorphic fungus *Beauveria bassiana *(Bals.) Vuill. (teleomorph: *Cordyceps bassiana*) is the most widely used mycopesticide for the biological control of insect pests [1,2], formulations based on this fungus being available for commercial use [3]. However, there are still many unresolved questions in our understanding of the life of fungal entomopathogens, including their population characteristics and relationships between genotypes and habitats or host-pathogen interactions [4]. For predictable and successful application of biological control agents (BCAs) to control diseases and pests in natural environments, their biology and ecology must be well understood [5-7].

The morphological features of conidia are common tools for identification in *Beauveria*. Morphological and molecular studies have shown that the broad patterns of diversity in *Beauveria *have been accurately predicted in previous morphological studies. However, they have also shown that these approaches are insufficient to investigate species such as *B. bassiana *[8]. Molecular data applied to taxonomic investigations have demonstrated that *B. bassiana *is a species complex with several cryptic species and have corroborated their link to *Cordyceps *teleomorphs [8-12]. In this sense, phylogenetic studies based on nuclear ITS and elongation factor 1-alpha (EF1-α) sequences have demonstrated the monophyly of *Beauveria *and the existence of at least two lineages within *B. bassiana s.l*. (*sensu lato*), and also that EF1-α sequences provide adequate information for the inference of relationships in this genus [8]. Studies on the genetic variability of BCAs such as *B. bassiana *are crucial for the development of molecular tools for their monitoring in the natural environment [6].

Minisatellite loci [13], random amplified polymorphism DNA (RAPD) [14], universally primed (UP) PCR [15], amplified fragment length polymorphism (AFLP) [16], isoenzyme analyses [17], or combinations of these methods [18] have provided useful polymorphisms to access genetic diversity among *B. bassiana *isolates.

Although some molecular studies have correlated *B. bassiana *genetic groups and host affiliation [9,19], more recent evidence indicates that this is not the case since *B. bassiana *contains generalist enthomopathogens with no particular phylogenetic association with their insect host [7,18], environmental factors being the prime selective forces for genotypic evolution in *B. bassiana *[7]. In this sense, several studies have demonstrated the association between *B. bassiana *genetic groups and Canadian [20], Brazilian [18] and world-wide [21] climatic zones.

Entomopathogenic species displayed a high degree of variability-mainly attributed to the presence of group I introns- at specific sites of the coding regions of small and large subunits of nuclear ribosomal RNA genes (SSU rDNA and LSU rDNA). Group I introns in entomopathogenic fungi were initially reported in *Beauveria brongniartii *LSU genes [22]. Work addressing the presence and usefulness of these non-coding elements has been reported for *Beauveria*. For example, Neuvéglise et al. [23] found 14 form variants of introns, differing in size and restriction patterns, at four different LSU positions from among a panel of 47 isolates of *B. brongniartii*, two of *B. bassiana*, and one of *Metarhizium anisopliae *from several geographic origins. Coates et al. [24] found 12 intron forms in the SSU from 35 *Beauveria *isolates. Wang et al. [25] analyzed the presence of group I introns in the four LSU insertion positions, designated Bb1 (also known as Ec2563), Bb2 (Ec2449), Bb3 (Ec2066) and Bb4 (Ec1921), and distributed a collection of 125 *B. bassiana *isolates in 13 different genotypes. In that study, their sequence analyses confirmed that the introns were invariably inserted in specific target sequences, and a strong correlation between specific insertion sites and intron subgroups was also observed. In addition, the features and behaviour of these group I intron were also detected in related genera such as *Cordyceps *[26] and *Metarhizium *[27].

The present study was undertaken to investigate the genetic variability existing in a collection of 53 Spanish isolates of *B. bassiana*, obtained from different substrates or insect hosts, and 4 isolates from other European countries. The insertion patterns of group I introns at the 3'-end of the LSU rDNA genes and EF1-α phylogenetic distribution were integrated in order to explore any possible correlation between genetic groups and geographical/climate origin, and habitat or insect host.

## Results

### Analysis of group I introns in 3' LSU rDNA

The 3'-end of the nuclear LSU rDNA genes of the 57 *B. bassiana *isolates (Table 1) was amplified with primers I29 and M1 and four different sizes of PCR products were observed on agarose gels, ranging from 0.79 to 1.77 kb. The sizes were as follows: about 1650 bp for 44 isolates; 1770 bp for one isolate; 1280 bp for 9 isolates, and 790 bp for 3 isolates. All amplicons were purified and sequenced in order to determine whether the insertion of multiple sequences, a feature described for members of this and other entomopathogenic genera, was responsible for the diversity of their lengths.

**Table 1:** Information concerning the *Beauveria bassiana *isolates analyzed in this study.

Code	Isolate	Location	Climate	Habitat/Host (Order)
Bb1	EABb 01/145-Su	Sevilla (Spain)	M	olive
Bb2	EABb 01/160-Su	Huelva (Spain)	M	oak
Bb3	EABb 01/164-Su	Huelva (Spain)	M	pine
Bb4	EABb 01/168-Su	Huelva (Spain)	M	scrubland
Bb5	EABb 01/171-Su	Huelva (Spain)	M	cotton
Bb6	EABb 01/15-Su	Almería (Spain)	M	dessert
Bb7	EABb 01/126-Su	Cádiz (Spain)	M	olive
Bb8	EABb 01/75-Su	Almería (Spain)	M	seaside
Bb9	EABb 01/116-Su	Sevilla (Spain)	M	olive
Bb10	EABb 01/112-Su	Sevilla (Spain)	M	wheat
Bb11	EABb 01/125-Su	Cádiz (Spain)	M	fallow land
Bb12	EABb 00/10-Su	Jaén (Spain)	M	olive
Bb13	EABb 00/11-Su	Jaén (Spain)	M	scrubland
Bb14	EABb 00/13-Su	Jaén (Spain)	M	woodland
Bb15	EABb 00/16-Su	Almería (Spain)	M	scrubland
Bb16	EABb 00/17-Su	Almería (Spain)	M	dessert
Bb17	EABb 01/07-Su	Córdoba (Spain)	M	meadow
Bb18	EABb 01/19-Su	Granada (Spain)	M	wheat
Bb19	EABb 01/22-Su	Córdoba (Spain)	M	scrubland
Bb20	EABb 01/25-Su	Córdoba (Spain)	M	olive
Bb21	EABb 01/27-Su	Córdoba (Spain)	M	wheat
Bb22	EABb 01/33-Su	Cádiz (Spain)	M	olive
Bb23	EABb 01/34-Su	Málaga (Spain)	M	olive
Bb24	EABb 01/35-Su	Málaga (Spain)	M	scrubland
Bb25	EABb 01/36-Su	Málaga (Spain)	M	meadow
Bb26	EABb 01/37-Su	Málaga (Spain)	M	olive
Bb27	EABb 01/43-Su	Jaén (Spain)	M	olive
Bb28	EABb 01/45-Su	Jaén (Spain)	M	scrubland
Bb29	EABb 01/64-Su	Granada (Spain)	M	woodland
Bb30	EABb 01/73-Su	Granada (Spain)	M	scrubland
Bb31	EABb 01/76-Su	Granada (Spain)	M	scrubland
Bb32	EABb 01/100-Su	Sevilla (Spain)	M	olive
Bb33	EABb 01/103-Su	Sevilla (Spain)	M	woodland
Bb34	EABb 01/105-Su	Sevilla (Spain)	M	cotton
Bb35	EABb 01/130-Su	Cádiz (Spain)	M	pine
Bb36	EABb 01/132-Su	Cádiz (Spain)	M	cotton
Bb37	EABb 90/2-Dm	Badajoz (Spain)	M	*Dociostaurus maroccanus *(Orthoptera)
Bb38	EABb 90/4-Cb	Badajoz (Spain)	M	*Chortipus bicolor *(Orthoptera)
Bb39	EABb 91/6-Ci	Badajoz (Spain)	M	*Calliptamus italicus *(Orthoptera)
Bb40	EABb 91/7-Dm	Badajoz (Spain)	M	*D. maroccanus *(Orthoptera)
Bb41	EaBb 92/10-Dm	Badajoz (Spain)	M	*D. maroccanus *(Orthoptera)
Bb42	EABb 92/11Dm	Badajoz (Spain)	M	*D. maroccanus *(Orthoptera)
Bb43	EABb 93/14-Tp	Córdoba (Spain)	M	*Thaumetopea pytiocampa *(Lepidoptera)
Bb44	EABb 04/01-Tip	Sevilla (Spain)	M	*Timaspis papaveris *(Hymenoptera)
Bb45	EABb 01/88-Su	South Portugal	M	sunflower
Bb46	EABb 01/39-Su	Málaga (Spain)	M	almond
Bb47	EABb 01/110-Su	Sevilla (Spain)	M	holm oak
Bb48	EABb 04/06-Su	Córdoba (Spain)	M	cork oak
Bb49	EABb 04/08-Su	Córdoba (Spain)	M	hazel
Bb50	EABb 04/02-Su	Santander (Spain)	HO	Ebro river
Bb51	EABb 04/03-Su	Santander (Spain)	HO	grassland
Bb52	EABb 04/05-Su	Álava (Spain)	C	leek
Bb53	EABb 04/09-Su	Madrid (Spain)	C	grassland
Bb54	EABb 04/10-Su	Gerona (Spain)	M	olive
Bb55	EABb 04/12-Su	Georgia	C	inculto
Bb56	*B. bassiana *1333	Greece	M	*Bactrocera oleae *(Diptera)
Bb57	*B. bassiana *3395	Poland	C	No data available

Code: reference as each isolate is cited in the text.

Source: reference as received from the Collection from the Department of *Ciencias y Recursos Agrícolas y Forestales (CRAF*) of the University of Córdoba, Spain.

Climatic: zones where isolates were collected (M: subtropical Mediterranean, C: continental, HO: humid oceanic).

After sequencing analysis (Table 2), we observed that the smallest PCR products were detected in 3 out of the 57 isolates studied-coded Bb19, Bb50 and Bb57- indicating that these isolates had no introns, and the intronless sequence size was 790 bp; identical in composition to a homologous fragment of *B. bassiana s.l*. [25] described previously. The other 54 isolates exhibited introns inserted at one or more of the four possible conserved positions. Among these 54 intron-containing isolates, the insertion was as follows: 44 showed inserted sequences at positions 1 (Ec2563) and 4 (Ec1921); one isolate, Bb51, with a sequence size of 1770 bp, contained two introns at positions 2 (Ec2449) and 4 (Ec1921), and nine isolates contained only one intron at position 4.

**Table 2:** Genotypes derived from the presence/absence of introns in LSU rDNA genes for 57 *Beauveria bassiana *isolates and types of intron sequences.

			GenBank		
Genotype * (%)	Isolate code	No. isolates	position 1 (Ec2563)	position 2 (Ec2449)	position 4** (Ec1921)
**A1B2B3A4**	Bb2-5, Bb32-33, Bb35, Bb45, Bb48-49	10	EF115312		EF115308 (433a)
	Bb1, Bb6-12, Bb14-17, Bb20-21, Bb23-31,				
	Bb34, Bb36, Bb41-42, Bb44, Bb46-47,				
	Bb52-54, Bb56	34	EF115312		EF115307 (443b)
**B1A2B3A4**	Bb51	1		EF115313	EF115309 (427)
**B1B2B3A4**	Bb13, Bb18	2			EF115310 (443c)
	Bb22, Bb37, Bb39-40, Bb43	5			EF115311 (443d)
	Bb38, Bb55	2			EF115309 (427)
**B1B2B3B4**	Bb19, Bb50, Bb57	3	-	-	-

*A, presence, and B, absence of a given intron at the 3'-end of the nuclear LSU rDNA genes. Numbers 1-4 represent insertion sites Ec2563, Ec2449, Ec2066 and Ec1921, respectively, as previously described [25].

** Sequence types inserted at position 4 (Ec1921) are indicated by their sizes in bp, followed by a letter for identical sizes (i.e., 443a, b, c, d) to indicate small differences in their composition.

The presence/absence of introns at the 3'-end of the nuclear LSU rDNA of the 57 isolates analyzed allowed their distribution in the following genotypes: A1B2B3A4, B1A2B3A4, B1B2B3A4 and B1B2B3B4 (A = presence, B = absence; according to Wang et al. [25]). Insertion sites are numbered from 1 to 4, also following Wang's terminology [25]: Ec2563 (position 1), Ec2449 (position 2), Ec2066 (position 3) and Ec1921 (position 4). These genotypes and their distribution frequencies are shown in Table 2. Three out of the 57 isolates had no introns; nine contained one, and forty-five had two introns. Fifty-four of 57 isolates showed an inserted intron at position 4, and 44 isolates at position 1, whereas only one isolate had an inserted intron at position 2. None of the 57 isolates had introns at the 3 insertion site.

There was a significant correlation between belonging to an intron genotype and the mean of the optimal (F_1,84_: 57.20°C; P < 0.001) and highest (F_1,84_: 27.39°C; P < 0.001) growth temperatures, which were significantly lower in the genotype B1B2B3A4, with T_opt _and T_max _values of 24.3 and 33.9°C, respectively, than those obtained for A1B2B3A4, with T_opt _of 26.7 and T_max _35.6°C (data not shown).

Two different intron sequence sizes, 427 or 443 bp in length, were detected at position 4 within the 54 *Beauveria *isolates that bore an insertion at this site, allowing the distribution of the isolates into two sub-genotypes (Table 2). Three of these 54 isolates had a sequence of 427 bp, showing 100% identity with the 4-position intron sequence reported for *B. bassiana *Bb232 [25]. In 51 of the *B. bassiana *isolates, the inserted sequence length at this position was 443 bp, and four variants with few nucleotide differences were observed after alignment of these sequences, showing identity values of 98 to 100% with another sequence detected at the same position in *B. bassiana *Bb726 [24].

The intron sequence inserted at position 2 was only detected for Bb51, an isolate obtained in Santander (North Spain), and was 502 bp long. This intron shared 99 and 98% identity with two sequences previously detected at the same position in the LSU of *B. bassiana *isolates 178 and 1121 [24,25]. A 387-bp intron was identified in 44 isolates at position 1. Alignment of these sequences revealed that the 387-bp sequence was conserved in the 44 *B. bassiana *isolates, where this intron was observed, and this sequence had identity values of 98% with the previously described sequence of *B. bassiana *ECBL16 [24].

The seven different *B. bassiana *intron sequences exhibited the typical characteristics of group I and no ORFs were detected. These intron sequences from *B. bassiana *were compared with other fungal intron sequences available in databases for their placement in previously reported subgroups [28]. The introns inserted at positions 2 and 4 were placed in the IC1 subgroup (one of the 15 subgroups, based on their secondary structure, described within the group I introns), and that inserted at position 1 was placed in the IE subgroup. As previously observed in group I introns [25-27], those inserted at the same site all belonged to the same subgroup. The intron sequences obtained in this work were compared with other *B. bassiana *intron sequences representing different subgroups to examine their polymorphisms (data not shown). Intron size and nucleotide identity differences were observed but P, Q, R and S motif elements, which are needed for the formation of the secondary structure of group I introns [29], were highly conserved among the introns inserted at the same site, particularly for position 1. The highest polymorphism was observed in introns inserted at 2, the P1-P3 helices being the source of this variation, and at 4, in the P5, P6 and P8 helices.

**Figure 1:**
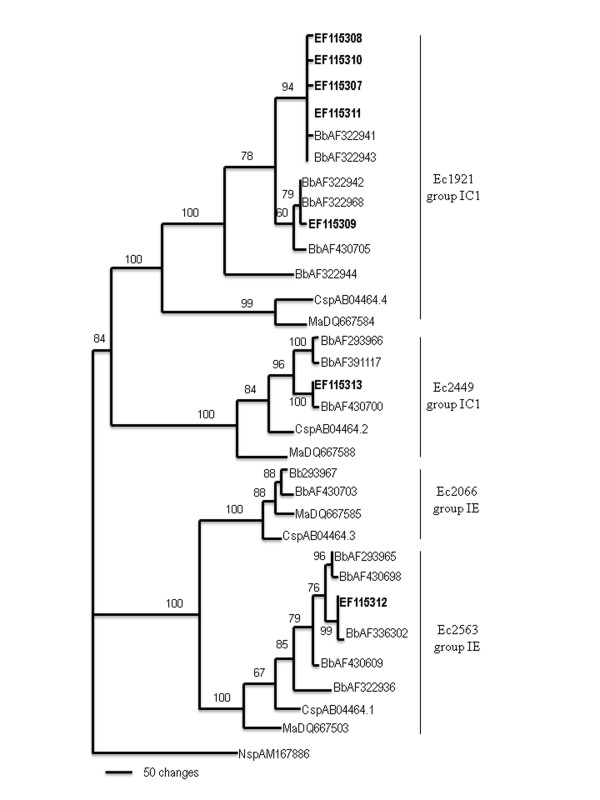
**Phylogenetic analysis of group I introns inserted in the LSU rDNA genes of entomopathogenic fungi**. The MP tree was generated by parsimony analysis after heuristic searches (TBR option). A bootstrap full heuristic analysis, with bootstrap intervals from 1000 replications and nodes supported in >50% of bootstrap replicates, was generated using the PAUP 4.0 program. Branch lengths are proportional to the number of changes. Seven different intron sequence types (bolded) identified from 57 *B. bassiana *isolates were aligned with 24 representative intron sequences from *Metarhizium anisopliae *(Ma), *Beauveria bassiana *(Bb) and *Cordyceps profilica *(Csp), and an intron sequence from *Naegleria *sp. (Nsp) was used as outgroup. The four group I intron insertion positions are shown as Ec1921 (position 4), Ec2066 (position 3), Ec2449 (position 2) and Ec2563 (position 1).

The MP tree obtained after an alignment of the 7 different intron sequence types identified from 57 *B. bassiana *isolates and another 24 GenBank-deposited sequences, which represent intron sequences from *M. anisopliae*, *B. bassiana *and *Cordyceps profilica*, together with the subsequent phylogenetic analysis are shown in Figure 1. The tree reveals the separation of four independent groups, supported by high bootstrap values, corresponding to the four positions reported previously [25]: Ec1921 (position 4), Ec2066 (position 3), Ec2449 (position 2) and Ec2563 (position 1), where intron insertions occurred. The tree shows that the sequence group located at position 4 is closer to those at position 2 and both contain IC1 subgroup introns. Similarly, position 3 sequences are closer to position 1 sequences, and both groups have IE subgroup introns. Within position 4, *Cordyceps *and *Metarhizium *were separated from *Beauveria *sequences and formed an independent group, supported by a bootstrap value of 100%. In addition, the five different *Beauveria *sequences obtained here were separated into two of the four observed groups at this position, supported by bootstrap values of 94% and 60%. This separation was in accordance with the two sequence sizes detected: 443 and 427-bp in length. However, the four different sequence types detected for 443-bp-sized introns were not separated after phylogenetic analysis.

### EF1-α gene analysis

**Figure 2:**
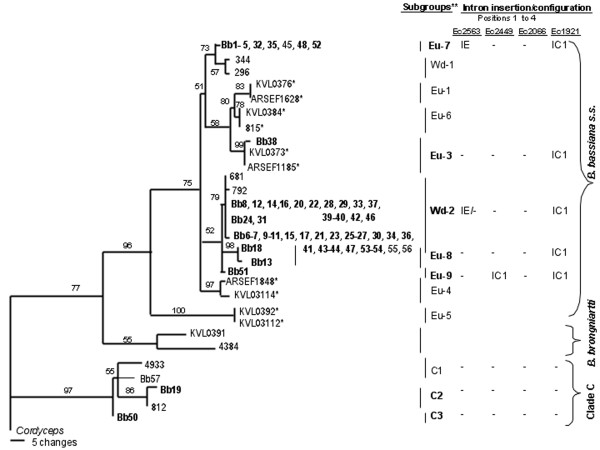
**Phylogenetic analysis based on EF1-*a *sequences from *Beauveria bassiana***. The MP tree was generated by parsimony analysis after heuristic searches (TBR option). A bootstrap full heuristic analysis, with bootstrap intervals from 1000 replications and nodes supported in >50% of bootstrap replicates, was generated using the PAUP 4.0 program. Branch lengths are proportional to the number of changes. Eleven sequence types identified from 56 *B. bassiana *isolates, of which 52 were sampled in Spain (bolded), were aligned with 18 GenBank *B. bassiana s.s., B. brongniartii *and *B. cf. bassiana *(clade C) sequences, indicated by accession numbers as in previous works [7,8]. *B. bassiana s.s*. EF1-α sequences representing European subgroups [7] are marked with an asterisk. Reference isolates from countries different to Spain, are referred to as: Eu-1 (KVL0376 from Denmark and ARSEF1628 from Hungary), Eu-3 (KVL0373 from Denmark and ARSEF1185 from France), Eu-4 (KVL03114 from Denmark and ARSEF1848 from Belgium), Eu-5 (KVL0392 and KVL03112 from Denmark), Eu-6 (KVL0384 from Denmark and 815 from France), Eu-7 (Bb45 from Portugal), Wd-1 (296 and 344 from USA), Wd-2 (681 from Romania, 792 from USA, Bb55 from Georgia and Bb56 from Greece), C1 (4933 from France and Bb57 from Poland), C2 (812 from France) and *B. brogniartii *(KVL0392 from Denmark and 4384 from China). *Cordyceps cf. scarabaeicola *(EFCC 2533) was the outgroup. Identifiers of EF1-α subgroups and intron configuration patterns are indicated.

With the exception of isolate Bb49, where no amplification was observed, all isolates afforded PCR products of 1.1 kb for the EF1-α gene with the primers tef1fw and 1750-R. Eleven different EF1-α gene sequences were identified among the 56 isolates. The alignment and comparison of these 11 sequences and another 18 GenBank-deposited sequences, representing different lineages from *B. bassiana s.s*. (*sensu stricto*), *B. brongniartii *and *B. bassiana *clade C [7,8,12], produced 1757 aligned positions, with 1542 constant characters and 114 parsimony-informative characters. The MP tree is shown in Figure 2. Of the 56 isolates analyzed, 94.6% (53 isolates) were located in the *B. bassiana s.s*. clade, and 5.4% (3 isolates) in clade C, which includes *B. cf*. (uncertain taxonomy) *bassiana *isolates. Within *B. bassiana s.s*., the 53 isolates analyzed in this study were separated in five subgroups (Eu-7, Eu-8 and Eu-9 with isolates from Spain and Portugal; Eu-3 from Spain, France and Denmark; and Wd-2 with world-wide distribution), supported by bootstrap values higher than 50%.

### Integration of intron insertion patterns and EF1-α phylogenetic distribution

In order to assess the phylogenic distribution of the different intron configuration types, they were mapped on the EF1-α tree (Figure 2). All 53 *B. bassiana s.s*. isolates showed an intron IC1 inserted at position 4. However, the IE intron inserted at position 1 was only present in the 10 isolates from subgroup Eu-7 and 33 out of 39 isolates from subgroup Wd-2. In particular, this subgroup included most of the Spanish isolates of *B. bassiana *forming an EF1-α phylogenetic group with isolates 681 from Romania and 792 from the USA [8] but displaying two different intron insertion models. Bb51 showed a unique intron insertion pattern, with an IC1 intron at position 2, and located separately in the Eu-9 subgroup. No introns were detected at any position in the three *B. cf. bassiana *isolates from clade C.

No correlation between EF1-α phylogenetic groups and insect host was observed. Although Eu-7 subgroup did not included isolates of insect origin, the Wd-2 subgroup grouped isolates collected from Diptera, Hymenoptera, Lepidoptera and Orthoptera. Moreover, Wd-2 isolates from Orthoptera displayed different intron insertion models (i.e., Bb37, Bb39 and Bb40, and Bb42).

Forty-nine Spanish and one Portuguese isolates of *B. bassiana s.s*. were collected from subtropical Mediterranean climate zones and were distributed in the Eu-7, Eu-3, Wd-2 and Eu-8 subgroups. Two Spanish isolates, Bb52 and Bb53, were collected from continental climate locations and were placed within subgroups Eu-7 and Wd-2, respectively. The only *B. bassiana s.s*. isolate from a humid oceanic climate included in this work, Bb51 from Santander, displayed a characteristic intron insertion model and formed the EF1-α subgroup Eu-9. In addition, Bb51 produced smaller conidia than the rest of *B. bassiana *isolates, this morphological feature being statistically significant (data not shown). Nevertheless, other isolate from the same climatic zone, Bb50, was grouped with other European isolates in *B. cf. bassiana *clade C.

## Discussion

In the present study, we have identified different *B. bassiana *genotypes and phylogenetic subgroups in a collection of 57 isolates of this fungus, based on intron insertion patterns and EF1-α phylogenies, respectively.

The variability in group I introns from rDNA genes has been used as a molecular tool for the identification of polymorphisms in entomopathogenic fungi [23,30,31]. Our study of *B. bassiana *LSU rDNA identified 99 introns among the 57 isolates analyzed. Four specific sites of intron insertion have been described previously in *Beauveria *species [23,25], but in our collection introns were only detected at positions 1, 2 or 4.

Particularly, our study shows that 100% of *B. bassiana s.s*. isolates had an intron inserted at position 4. This position was also preferential for intron insertion (84.4%) in a population of 125 *B. bassiana *isolates [25]. The number of introns found in the 57 isolates was in agreement with the 199 introns detected in 125 *B. bassiana *isolates by Wang et al. [25]; the 44 introns detected in 26 *M. anisopliae *isolates by Márquez et al. [31], and the 69 introns found in 28 representative members of the genus *Cordyceps *by Nikoh and Fukatsu [26]. However, only four intron insertion patterns were present in our *B. bassiana *collection while greater variability was found in other studies: 13, 7 and 9 insertion patterns within 125 *B. bassiana *[25], 26 *M. anisopliae *[31] and 47 *B. brongniartii *[23] isolates, respectively.

The MP tree based on intron sequences shows that they were distributed in four large groups, with bootstrap values of 100%, corresponding to four insertion positions (Figure 1). As could be expected [25,28], the introns inserted at the same site always belonged to the same subgroup: IC1 at positions 2 and 4, and IE at position 1. Although the origin and transmission mechanisms of group I introns have generated controversy [26], this distribution of sequences is in agreement with previously reported observations [25] and means that introns inserted at the same position have a monophyletic origin and are transmitted vertically. In subsequent events intron speciation and diversification take place as occurs at position 4, where *B. bassiana *introns are separated from *Metarhizium *and *Cordyceps *introns, and two *B. bassiana *IC1 sequence sizes were located in two different sub-clades, supported by high bootstrap values.

Rehner and Buckley's study [8] based on EF1-α and ITS phylogenies has revealed that i) six clades can be resolved within *Beauveria *(A-F) and, excepting those corresponding to *B. bassiana *(A and C), they are closely to species previously described on the basis of their morphology, and ii) *B. bassiana s.s*. (A) was determined almost entirely from nucleotide variation at EF1-α. Further phylogenetic studies carried out with nuclear and/or mitochondrial DNA regions of *B. bassiana *from all continents have served to resolve lineage diversity within this species [7,12,18,21]. Since phylogenetic species by continent and in the order of their discovery have been designated previously [7], we followed this nomenclature to refer the new phylogenetic subgroups identified among the Spanish *B. bassiana s.s*. isolates as Eu-7, Eu-8 and Eu-9. The results obtained from MP analyses (Figure 2), using a 1.1 kb fragment of the EF1-α gene from 56 isolates from our collection, confirmed that 53 isolates were *B. bassiana s.s*. (A), and three isolates grouped in three different phylogenetic subgroups within *B. cf. bassiana *(C). As in a previous study [7], the collection of Spanish isolates of *B. bassiana s.s*. was separated in five phylogenetic subgroups. However, only isolate Bb38, sampled from insects, was grouped with one (Eu-3) of the five phylogenetic species proposed by those authors working with a Danish collection of *B. bassiana s.s*. [7], including insect isolates only. Interestingly, three phylogenetic subgroups (Eu-7, Eu-8 and Eu-9) were only formed by isolates from Spanish and Portuguese isolates. However, most of the isolates in our collection (39 out of 56) were grouped with isolates from Romania and the USA in the world-wide phylogenetic subgroup Wd-2, which includes isolates from Europe, Africa and North America [8].

When the different intron insertion patterns were mapped on the *B. bassiana *EF1-α phylogeny (Figure 2), the existence of a same intron genotype in a given phylogenetic subgroup could be indicative of its clonal origin as it is the case of Eu-7 and Eu-8. Previous studies have shown that Eu-3, where Bb38 is located, is a clonal group [7]. Isolate Bb51 was the only member of Eu-9 and the separated phylogenetic grouping of this isolate is supported by a characteristic intron insertion pattern and the production of statistically significant smaller conidia than those from any other intron genotype (data not shown). The two different intron genotypes observed among the isolates from the complex phylogenetic subgroup Wd-2, may indicate that homologous recombination is involved in the IE intron loss at position 1. Previous studies have shown frequent intron losses of group I introns in the nuclear rDNAs of *Cordyceps *[26]. Recently, a low frequency of sexual reproduction was observed in Eu-1 [7]; this could also be the case of Wd-2 where the absence of an IE intron at position 1 was only observed in 6 out of 39 isolates of this phylogenetic subgroup.

The genetic diversity of Spanish *B. bassiana s.s*. isolates was compared in relation to their hosts and geographical provenance and according to the latter view [21], no general correlation can be observed between the molecular variability among isolates and host and/or geographical origin. Although most of the isolates in our study were collected from soil, 8 out of 9 isolates from insects were grouped together in the subgroup Wd-2 although they derived from different insect orders. Phylogenetic subgroups only indicated a tenuous dependence upon geographic origin (i.e., Bb2-5 located in Eu-7 or Bb23-26 and Bb29-31 located in Wd-2). A recent phylogeographic report [18] has provided evidence that the genetic distance of Brazilian *B. bassiana *isolates correlates with geographical distance, suggesting that according to Rehner's study [12] allopatry plays an important role in the phylogenetic diversification of *B. bassiana*. The authors of another recent study [7] concluded that multiple phylogenetic species of *B. bassiana s.s*. co-exist in sympatry within the limited natural habitat of a bordering hedgerow. We observed that isolates sampled in close locations were placed in different phylogenetic subgroups (i.e., Bb35 and Bb36, from Cádiz, belong to Eu-7 and Wd-2, respectively; and Bb38, and Bb39-40 and Bb42, from Badajoz, group within Eu-3 and Wd-2, respectively, Bb39-40 and Bb42 having different intron genotypes). According to Meyling's study [7], the high phylogenetic diversity of the Spanish isolates of *B. bassiana s.s*. could be explained by the untilled habitats where most of them were sampled (i.e., olive, oak, pine, meadow or scrubland).

Previous studies have suggested that the saprophytic phase of entomopathogenic fungi exerts evolutionary pressure on the genotype and that adaptation to a habitat type is associated with their environmental preferences [20]. Recent studies have also pointed out the importance of climatic conditions in the prevalence and distribution of *B. bassiana *genotypes [21]. Our study was carried out on 51 isolates from subtropical Mediterranean climate locations that were distributed within the phylogenetic subgroups Eu-3, Eu-7, Eu-8, Wd-2 and clade C; 4 isolates were from continental climate sites and grouped in Eu-7, Wd-2 and clade C; and 2 isolates came from a humid oceanic climate zone, being located in Eu-9 and clade C. Interestingly, the only *B. bassiana s.s*. from a humid oceanic climate was the singular isolate Bb51. The fact that isolates from Mediterranean or continental climates overlapped in different phylogenetic subgroups, could be due to lower differences among the abiotic conditions existing in Spain, a country covering far smaller geographical surface and with much less variability than that considered in other Canadian, Brazilian or world-wide studies where phylogenetic species showed a better correlation with climate characteristics [21], biogeographic distribution [18] and habitat [20]. In a thermal growth study [20] it was described that *B. bassiana *genetic groups from three different habitats in Canada were associated with temperature preferences. When we explored the thermal preferences within a set of Spanish *B. bassiana s.s*. isolates belonging to the two main intron genotypes (A1B2B3A4 and B1B2B3A4) and four phylogenetic EF1-α subgroups (data not shown), a correlation between intron genotypes and the mean optimal and maximum temperatures for growth was observed, both growth temperatures being significantly lower in the B1B2B3A4 genotype with respect to A1B2B3A4. However, no correlation was observed between thermal preferences and the climatic origin of the Spanish *B. bassiana *isolates.

## Conclusion

Four intron genotypes, and five and three phylogenetic subgroups within *B. bassiana s.s*. and *B. cf. bassiana *(clade C) have been identified, respectively, in a collection of 57 *B. bassiana *isolates -53 from Spain. The highest polymorphism was observed in introns inserted at positions 2 and 4. All *B. bassiana s.s*. displayed an IC1 intron inserted at position 4. Integration of intron insertion patterns and EF1-α phylogenetic distribution served to demonstrate the monophyletic origin and vertical transmission of introns inserted at the same site. In subsequent events intron speciation and diversification take place as occurs at site 4, where *B. bassiana *introns are separated from *Metarhizium *and *Cordyceps *introns. No general correlation was observed between the molecular data and insect host, but a tenuous correlation was detected with the geographic origins. The high phylogenetic diversity of the Spanish isolates of *B. bassiana s.s*. could be due to the untilled habitats where most of them were sampled.

## Methods

### Fungal isolates and morphological studies

The 57 isolates of *B. bassiana *used in this study were selected from a Spanish collection of 960 records at the CRAF (*Ciencias y Recursos Agrícolas y Forestales*) Department of the University of Cordoba (Córdoba, Spain), representing different geographic origins, habitats/hosts and climates. Fifty-three Spanish isolates were studied, 51 of them being collected from subtropical Mediterranean climate zones -characterized by warm to hot, dry summers and mild to cool, wet winters- and 2 from a humid oceanic climate. Forty-five out of these 53 isolates were from soil, most of them from poorly tilled or untilled fields (i.e., olive, oak, pine or scrubland) and 8 were isolated from insects. Information about these isolates is provided in Table 1. All fungal isolates were derived from single conidial spores grown on Malt Extract Agar plates (MEA, Difco Becton Dickinson, Sparks, MD).

### DNA extraction, PCR amplification, and sequencing

Mycelia for DNA extraction were obtained as previously described [31]. Total DNA was extracted using the method previously described [32].

Two nuclear gene regions, LSU rDNA and EF1-α, were amplified, sequenced and analyzed. The 3'-end of the nuclear LSU rDNA cluster was also amplified with primers I29 (5'-CTGCCCAGTGCTCTGAATGTC-3') [25] and M1 (5'-GGTAAAACTAACCTGTCTCACG-3') [31] for the 57 isolates of *Beauveria *included in the study. The distribution of putative introns was investigated using the following combinations of previously described primers: I29-I38, I31-I32, I21-I22 and E23-M1 [25,31]. A 1100 bp fragment spanning the 3' 2/3 of the EF1-α gene was amplified with primers tef1fw (5'-GTGAGCGTGGTATCACCA-3') [33] and 1750-R (5'-GACGCATGTCACGGACGGC-3') for all isolates, except Bb49. The oligonucleotide 1750-R was designed at the 3'-end of an alignment of *Beauveria *EF1-α genes obtained from databases. PCR was performed in a total volume of 50 μl containing 25 ng of genomic DNA and 0.20 μM concentrations of each of the above primers, using the *Taq *polymerase system (Biotools B&M Labs, Madrid, Spain) and following the manufacturer's instructions. The amplification program included an initial denaturing cycle of 1 min at 94°C, followed by 35 cycles of 1 min 30 s at 94°C, 2 min (for EF1-*a*) or 2 min 30 sec for (LSU rDNA) at 55 (for EF1-*a*) or 57°C (for LSU rDNA) and 3 min at 72°C, and a final extension step of 7 min at 72°C in a PCR System 9700 Genetic Thermal Cycler (Applied Biosystems, Foster City, CA). The PCR products were electrophoresed on 1% agarose gels buffered with 1 × TAE [34] and stained with ethidium bromide. A 100-bp ladder molecular weight standard (Roche Mannheim, Mannheim, Germany) was also used.

The PCR products were purified from agarose gels using the Geneclean II kit^® ^system (Q-Biogene, Carlsbad, CA), following the manufacturer's protocol. DNA sequences were obtained using an automated ABI 377 Prism Sequencer (Applied Biosystems, Foster City, CA) with fluorescent terminators at the Department of Microbiology and Genetics of the University of Salamanca. All PCR products were sequenced in both directions, using amplification primers and internal primers when necessary.

The intron and EF1-α sequences obtained in this study were deposited in the GenBank database. Intron and EF1-α sequence accession numbers are available in Table 2 and additional file 1 respectively.

### Molecular analyses

The presence or absence of introns at the 3'-end of the nuclear LSU rDNA of each isolate was determined by detecting previously described target sequences [25]. In order to compare the results obtained in this study with the *B. bassiana *genotypes based on previously reported intron insertion patterns in the LSU rDNA gene, Wang's terminology was used [25]. The intron sequences detected in each insertion point were aligned with representative *Beauveria *sequences to examine their polymorphisms and to identify conserved motifs. Intron subgroups were determined by comparison with representative secondary structures from previous studies [25-27,30].

Intron and EF1-α sequences were analyzed separately. Published sequences for isolates included within the genera *Beauveria*, *Metarhizium *and *Cordyceps *were retrieved from GenBank and included in the alignments. Alignments were generated using the MegAlign (DNASTAR package, 1989-92, London, UK) and the CLUSTALX 1.81 program [35]. Phylogenetic analyses were carried out with the PAUP* version 4.0 b10 program. Gaps, encoded as missing data, and uninformative characters were excluded from the analyses. Most-parsimonious (MP) trees were obtained for intron and EF1-α data from heuristic searches using TBR branch-swapping [36], and all MP trees were summarized in a single tree in which all branch lengths equal to zero were collapsed by polytomies. An intron sequence of *Naegleria *sp. (AM167886) and the EF1-α gene of *Cordyceps cf. scarabaeicola *(AY531967) were used as outgroups in the analysis of intron and EF1-α sequences, respectively. A bootstrap full heuristic analysis consisting of 1000 replicates was performed, and a 50% majority rule tree was produced.

## Authors' contributions

IGJ carried out the laboratory work related to EF1-α. MM and EAI carried out the laboratory work on introns. EQM and CSA provided the *B. bassiana *isolates. In addition, EQM and AOU participated in genomic DNA extraction. EM conceived the design of the study and helped to write the manuscript. RH participated in the design and coordination of the study, in the sequence analyses and wrote the manuscript. All authors have read and approved the final manuscript.

## Supplementary Material

Additional file 1 **Table of GenBank accession numbers of EF1-*a *sequences obtained in this study from 57 *Beauveria bassiana *isolates and EF1-α subgroups**. Click here for file
