# Supplementary material for: Genetic analyses place most Spanish isolates of Beauveria bassiana in a molecular group with word-wide distribution
Source: BMC Microbiol. 2011 Apr 26;11:84. doi: 10.1186/1471-2180-11-84 (PMC3111375; doi:10.1186/1471-2180-11-84)
Supplement: Additional file 1 — Table of GenBank accession numbers of EF1-a sequences obtained in this study from 57 Beauveria bassiana isolates and EF1-α subgroups. [file 1471-2180-11-84-S1.DOC]

GenBank accession numbers of EF1-*α* sequences obtained in this study from 57 *Beauveria bassiana* isolates and EF1-α subgroups

| Code^a^ | Isolate^b^ | EF1-α | EF1-α subgroup |
| --- | --- | --- | --- |
| Bb1 | EABb 01/145-Su | FJ545730 | Eu-7 |
| Bb2 | EABb 01/160-Su | FJ545730 | Eu-7 |
| Bb3 | EABb 01/164-Su | FJ545730 | Eu-7 |
| Bb4 | EABb 01/168-Su | FJ545730 | Eu-7 |
| Bb5 | EABb 01/171-Su | FJ545730 | Eu-7 |
| Bb6 | EABb 01/15-Su | FJ545732 | Wd-2 |
| Bb7 | EABb 01/126-Su | FJ545732 | Wd-2 |
| Bb8 | EABb 01/75-Su | FJ545731 | Wd-2 |
| Bb9 | EABb 01/116-Su | FJ545732 | Wd-2 |
| Bb10 | EABb 01/112-Su | FJ545732 | Wd-2 |
| Bb11 | EABb 01/125-Su | FJ545732 | Wd-d |
| Bb12 | EABb 00/10-Su | FJ545731 | Wd-2 |
| Bb13 | EABb 00/11-Su | FJ545740 | Eu-8 |
| Bb14 | EABb 00/13-Su | FJ545731 | Wd-2 |
| Bb15 | EABb 00/16-Su | FJ545732 | Wd-2 |
| Bb16 | EABb 00/17-Su | FJ545731 | Wd-2 |
| Bb17 | EABb 01/07-Su | FJ545732 | Wd-2 |
| Bb18 | EABb 01/19-Su | FJ545733 | Eu-8 |
| Bb19 | EABb 01/22-Su | FJ545735 | C2 |
| Bb20 | EABb 01/25-Su | FJ545731 | Wd-2 |
| Bb21 | EABb 01/27-Su | FJ545732 | Wd-2 |
| Bb22 | EABb 01/33-Su | FJ545731 | Wd-2 |
| Bb23 | EABb 01/34-Su | FJ545732 | Wd-2 |
| Bb24 | EABb 01/35-Su | FJ545739 | Wd-2 |
| Bb25 | EABb 01/36-Su | FJ545732 | Wd-2 |
| Bb26 | EABb 01/37-Su | FJ545732 | Wd-2 |
| Bb27 | EABb 01/43-Su | FJ545732 | Wd-2 |
| Bb28 | EABb 01/45-Su | FJ545731 | Wd-2 |
| Bb29 | EABb 01/64-Su | FJ545731 | Wd-2 |
| Bb30 | EABb 01/73-Su | FJ545732 | Wd-2 |
| Bb31 | EABb 01/76-Su | FJ545739 | Wd-2 |
| Bb32 | EABb 01/100-Su | FJ545730 | Eu-7 |
| Bb33 | EABb 01/103-Su | EF545731 | Wd-2 |
| Bb34 | EABb 01/105-Su | FJ545732 | Wd-2 |
| Bb35 | EABb 01/130-Su | FJ545730 | Eu-7 |
| Bb36 | EABb 01/132-Su | FJ545732 | Wd-2 |
| Bb37 | EABb 90/2-Dm | FJ545731 | Wd-2 |
| Bb38 | EABb 90/4-Cb | FJ545734 | Eu-3 |
| Bb39 | EABb 91/6-Ci | FJ545731 | Wd-2 |
| Bb40 | EABb 91/7-Dm | FJ545731 | Wd-2 |
| Bb41 | EaBb 92/10-Dm | FJ545732 | Wd-2 |
| Bb42 | EABb 92/11Dm | FJ545731 | Wd-2 |
| Bb43 | EABb 93/14-Tp | FJ545732 | Wd-2 |
| Bb44 | EABb 04/01-Tip | FJ545732 | Wd-2 |
| Bb45 | EABb 01/88-Su | FJ545730 | Eu-7 |
| Bb46 | EABb 01/39-Su | FJ545731 | Wd-2 |
| Bb47 | EABb 01/110-Su | FJ545732 | Wd-2 |
| Bb48 | EABb 04/06-Su | FJ545730 | Eu-7 |
| Bb49 | EABb 04/08-Su | Not available | Unknown |
| Bb50 | EABb 04/02-Su | FJ545736 | C3 |
| Bb51 | EABb 04/03-Su | FJ545737 | Eu-9 |
| Bb52 | EABb 04/05-Su | FJ545730 | Eu-7 |
| Bb53 | EABb 04/09-Su | FJ545732 | Wd-2 |
| Bb54 | EABb 04/10-Su | FJ545732 | Wd-2 |
| Bb55 | EABb 04/12-Su | FJ545732 | Wd-2 |
| Bb56 | *B. bassiana* 1333 | FJ545732 | Wd-2 |
| Bb57 | *B. bassiana* 3395 | FJ545738 | C1 |

^a^ Code: reference as each isolate is cited in the text.

^b^ Source: reference as received from the Collection from the Department of *Ciencias y Recursos Agrícolas y Forestales (CRAF)* of the University of Córdoba, Spain.
